# Country-specific data on the contraceptive needs of adolescents

**DOI:** 10.2471/BLT.16.189829

**Published:** 2017-03-01

**Authors:** Michelle J Hindin, Amanda M Kalamar

**Affiliations:** a Department of Reproductive Health and Research, World Health Organization, 20 Avenue Appia, 1211 Geneva 27, Switzerland.

The sustainable development goals (SDGs)^1^ and the *Global strategy for women’s, children’s and adolescents’ health 2016–2030*^2^ have placed renewed emphasis on understanding and addressing the sexual and reproductive health needs of adolescents. Although they are the largest cohort of adolescents in history, to date, we know relatively little about their contraceptive needs. This is particularly true for unmarried adolescents as data for them are rarely reported, even when collected. As the SDGs and the global strategy^2^ require country-level tracking of indicators related to contraception, including met need,^3^ a key question is “What can be done to support adolescents to prevent unintended pregnancy?”

To answer this question, we developed country-specific fact sheets^4^ describing adolescent contraceptive use and non-use in 58 low- and middle-income countries spanning all six World Health Organization Regions. The data in the fact sheets come from the Demographic and Health Surveys (DHS) Program,^5^ which routinely conducts nationally representative surveys in conjunction with country governments. All data in the fact sheets are publicly available.

To better understand the demographic situation of adolescents in each country, we provide information on the total number of adolescents,^6^ where they predominantly live (rural or urban),^6^ their average years of schooling, their average age at first birth, their average age at first union (i.e. married or living together) and their average age at first sex. Additionally, we provide estimates on the total number of male and female adolescents that are currently sexually active (i.e. reporting sex in the last three months or in a union) and the proportion of unmarried adolescents that are currently sexually active, disaggregated by sex. Taken together, these provide a crucial first step to understanding pregnancy risk, both within and outside unions at the country level.

To help policy-makers and programme managers understand the heterogeneity of adolescents’ contraceptive needs, we provide evidence to plan for how, when and where different groups of sexually active adolescents use and do not use contraception. Data on current 15–19 year old adolescent girls’ contraceptive use and non-use by method type are presented by marital status. Contraceptive methods include traditional (withdrawal or abstinence) and modern methods (spermicides, female condom, male condom, standard days/cycle beads, oral contraceptives, injectable contraceptives, lactational amenorrhea, implants, intrauterine device [IUD], male sterilization or female sterilization). Both within and across countries, there are significant variations in the types of methods used by adolescents – ranging from traditional methods to long acting reversible methods (implants and IUDs) and even to permanent methods in selected settings.

Understanding the varied reasons for non-use can help in tailoring policy and programme responses within countries to decrease barriers in knowledge and access for adolescents and health care providers. We report the top three reasons adolescent girls provide for why they are not currently using contraception, even though they do not want to become pregnant in the next two years. The data are based on responses from 15–19 year old adolescent girls, and are presented separately for those unmarried and sexually active and those in a union. Reasons for non-use vary considerably but among the most common reported are, being “not married” and infrequent sexual relations for unmarried, sexually active adolescents. In contrast, currently breastfeeding or postpartum abstinence are among the most common reasons for non-use reported by adolescents in a union. Fear of side-effects or health concerns was commonly reported by both groups of adolescent girls.

To provide a clearer picture of the potential sources of contraceptive commodities and help develop a robust set of high quality services for adolescents, it is essential to understand that adolescents get contraception from a variety of sources. We report on the two most common sources from which adolescents who are currently using a modern method most recently obtained that contraceptive method. The sources are driven by the types of contraceptive methods available, as well as those that are easy for adolescents to access. In some settings most sources are in the formal sector, including government facilities, private facilities and pharmacies. In other settings most adolescents obtain contraceptive commodities in the informal sector, such as shops, kiosks or roadside stands, or from friends. The data from the fact sheets indicate where best to target investments to improve access to – and quality of – contraceptive services for adolescents.

The data provided in these fact sheets are disaggregated by age and marital status to address the calls for ensuring that no one is left behind. These data can help policy-makers and programme planners reduce inequities in service provision and access, and to make evidence-based decisions about how to better address adolescents’ contraceptive needs.
